# Development of disk diffusion susceptibility test methods for *Aerococcus* spp. and updates to Clinical and Laboratory Standards Institute MIC breakpoints

**DOI:** 10.1128/jcm.00115-25

**Published:** 2025-05-14

**Authors:** Richard Maynard, Carmila Manuel, Synthia Simpkins, Michelle Haro, Nathan Ledeboer, Patricia J. Simner, Mark Fisher, Romney Humphries

**Affiliations:** 1 Department of Pathology, Microbiology and Immunology, Vanderbilt University Medical Center 204907, Nashville, Tennessee, USA; 2 Department of Pathology, Johns Hopkins University School of Medicine 198422 , Baltimore, Maryland, USA; 3 Department of Pathology, Medical College of Wisconsin 733705, Milwaukee, Wisconsin, USA; 4 Department of Medicine, Johns Hopkins University School of Medicine 229384 , Baltimore, Maryland, USA; 5 Department of Pathology and ARUP Laboratories, The University of Utah 161530, Salt Lake City, Utah, USA; Erin McElvania, Endeavor Health, Evanston, Illinois, USA

**Keywords:** *Aerococcus*, MIC, disk diffusion, CLSI, susceptibility testing

## Abstract

*Aerococcus* spp. are uropathogenic gram-positive bacteria that occasionally cause invasive infections. Minimal inhibitory concentration (MIC) breakpoints published by the Clinical and Laboratory Standards Institute (CLSI) were evaluated, and disk diffusion criteria developed, by testing 174 isolates of *Aerococcus* spp., by the CLSI M45 fourth edition workgroup. *Aerococcus urinae* growth was found to be significantly impacted by the brand of reference Mueller-Hinton broth base, and 14.6% of 117 *A*. *urinae* tested did not grow in cation-adjusted Mueller-Hinton broth supplemented with 5% lysed horse blood after 44 h incubation at 35°C in 5% CO_2_ in any brand. Ampicillin and nitrofurantoin MIC breakpoints were established, and penicillin MIC breakpoints were adjusted, based largely on MIC distributions and pharmacokinetic data. Disk criteria were established for penicillin, ampicillin, vancomycin, nitrofurantoin, ciprofloxacin, levofloxacin, and tetracycline. Disk breakpoints could not be established for cefotaxime, ceftriaxone, or meropenem using standard CLSI disk masses due to large zones of growth inhibition that did not differentiate between MIC values and error rates that far exceeded CLSI standards. Trimethoprim-sulfamethoxazole breakpoints were also not possible to establish due to differences in thymidine content between MIC and disk diffusion testing media, which led to no correlation between MIC values and zones of growth inhibition for this antibiotic. The data summarized herein are the basis for proposed changes to the M45 guideline for *Aerococcus* spp., which will be published in the fourth edition.

**IMPORTANCE:**

*Aerococcus* species are an increasingly recognized cause of urinary tract infections and may cause serious infections, including endocarditis. Antimicrobial susceptibility testing (AST) is required for the management of these infections, as species within the genus display resistance to various antimicrobial agents. This study describes the data that informed updates to the Clinical and Laboratory Standards Institute M45 guideline for AST of fastidious or infrequently isolated bacteria. We describe the introduction of new ampicillin and nitrofurantoin breakpoints, updated MIC breakpoints for penicillin, and disk diffusion correlates for penicillin, ampicillin, ciprofloxacin, levofloxacin, nitrofurantoin, and tetracycline.

## INTRODUCTION

*Aerococcus* spp. are catalase-negative gram-positive cocci, which grow in clusters. Historically misidentified as viridans group streptococci due to their colony morphology, the aerococci have increasingly been identified in urine and blood specimens following widespread introduction of matrix-assisted laser desorption ionization time-of-flight mass spectrometry (MALDI-TOF MS), a tool which has facilitated their identification. *Aerococcus urinae* is the most frequently isolated member of the genus, followed by *Aerococcus sanguinicola* and *Aerococcus viridans* (1, 2). Isolates identified as *A. urinae* were recently proposed to represent six unique taxa, including the named *A. tenax*, *A. mictus*, *A. loyolae,* and *A. urinae* (1),(3) but clinical laboratories have not widely adopted these new designations. Other members of the genus, *Aerococcus christensenii* and *Aerococcus urinaehominis,* are only occasionally recovered from human specimens and rarely, if ever, cause infections (2).

Data from a large retrospective study conducted in Sweden found aerococci were isolated in 0.4% of urine cultures (4). Retrospective review of these cases found 20%–25% were associated with asymptomatic bacteriuria. The remaining 75%–80% were associated with symptomatic urinary tract infections (UTI), including pyelonephritis in 8% of cases where *A. sanguinicola* was isolated and 18% of cases where *A. urinae* was isolated (4). These data reflect other reports in the literature on rates of recovery and pathogenicity (2). Aerococcal bacteriuria generally occurs in older patients, with median patient age across cases reported globally being 78–82 years of age (5–8). However, aerococci may be associated with UTI outside the elderly, with a recent report documenting cases of aerococcal UTI in pediatric patients, most of whom had a pre-existing urogenital disorder (9). Based on these data, *Aerococcus* spp. were added to the list of micro-organisms recognized as uropathogens by the European Federation of Clinical Chemistry and Laboratory Medicine (10). Specifically, *Aerococcus* spp. were defined as a secondary uropathogen species, i.e., a rare, but real, cause of primary infection in patients with a normal urinary tract. The European guidelines recommend performing both full identification and antimicrobial susceptibility testing (AST) of aerococci if isolated at ≥10^3^ CFU/mL in pure culture from urine specimens with white blood cell counts >30 × 10^6^/L and suggest optional AST if aerococci are recovered as part of a mixed culture (10). In contrast, the *Clinical Microbiology Procedures Handbook* highlights *A. urinae* as an emerging pathogen that requires species-level identification when isolated in significant quantities based on the urine source but does not advocate for AST to be performed (11).

Aerococci are also occasionally recovered from blood cultures. Estimates from Sweden suggest the incidence of aerococcal bacteremia is 14 cases per one million inhabitants; 6.5% of bacteremia cases in this study were associated with endocarditis (12). Similarly, a study conducted at the Mayo Clinic in the USA found that 49 of 219 cases of aerococcal bacteremia (22%) were clinically significant and 7 (3.2%, all *A. urinae*) were associated with endocarditis (13). In general, infective endocarditis caused by *A. urinae* occurs in older male patients with underlying urinary tract disease. Mortality estimates from endocarditis caused by *A. urinae* range widely from 0% to 45% (14). When isolated, experts suggest AST be performed to inform treatment decisions (12).

Both the Clinical and Laboratory Standards Institute (CLSI) and European Committee for Antimicrobial Susceptibility Testing (EUCAST) have developed AST methods for *Aerococcus* spp. The guidelines from both organizations are presented in Table 1 (15, 16). One limitation of the CLSI method is the lack of breakpoints for oral antimicrobial agents other than ciprofloxacin and levofloxacin, which should generally not be used for the treatment of uncomplicated cystitis (17). Furthermore, CLSI did not develop a disk diffusion method for *Aerococcus* spp. Gradient diffusion has not been validated for aerococci by commercial manufacturers, although laboratories have validated it for *A. urinae* with varying success (18, 19). While EUCAST has published a disk diffusion method, it employs Mueller-Hinton Fastidious agar media (MH-F) and disks with drug masses (i.e., antimicrobial agent content in each disk) that are not available in all countries, including the USA. This study describes the CLSI disk diffusion method and tentative breakpoints for *Aerococcus* spp. developed by the M45 guideline committee, for the fourth edition.

**TABLE 1:** Current CLSI and EUCAST guidelines for AST of *Aerococcus* spp.^*a*^

	This study	CLSI M45 third edition	EUCAST
MIC test method	CAMHB-LHB incubated at 35°C ± 2°C in 5% CO_2_ atmosphere 20–24 h incubation period	CAMHB-LHB incubated at 35°C ± 2°C in 5% CO_2_ atmosphere20–24 h incubation period	MH-F broth incubated at 35°C ± 1°C in ambient atmosphere 16–20 h incubation period, may incubate for a total of 40–44 h
Disk diffusion method	BMHA incubated at 35°C ± 2°C in 5% CO_2_ atmosphere 20–24 h incubation period		MH-F agar incubated at 35°C ± 1°C in 5% CO_2_ atmosphere 16–20 h incubation period, may incubate for a total of 40–44 h
Antimicrobial agents with breakpoints	Penicillin Ampicillin Amoxicillin Cefotaxime Ceftriaxone Meropenem Vancomycin Ciprofloxacin Levofloxacin Nitrofurantoin Tetracycline Trimethoprim-sulfamethoxazole	Penicillin Cefotaxime Ceftriaxone Meropenem Vancomycin Ciprofloxacin Levofloxacin Tetracycline Trimethoprim-sulfamethoxazole Linezolid	Benzylpenicillin Ampicillin Amoxicillin Meropenem Vancomycin Ciprofloxacin^*b*^ Levofloxacin^*b*^ Norfloxacin^*b*^ Nitrofurantoin Rifampicin

^*a*^ BMHA, blood Mueller-Hinton agar plates; CAMHB-LHB, cation-adjusted Mueller-Hinton broth supplemented with 5% lysed horse blood.

^*b*^ By EUCAST breakpoints, levofloxacin susceptibility is inferred from ciprofloxacin, and norfloxacin disk can be used as a screen for fluoroquinolone resistance.

## MATERIALS AND METHODS

### MIC data

MIC data were obtained from laboratories participating in the CLSI M45 workgroup, as well as those publicly available from EUCAST, on their website. Data were predominantly from one large reference laboratory (ARUP Laboratories, Salt Lake City, Utah), which performed testing using lyophilized, custom broth microdilution (BMD) panels obtained from ThermoFisher Scientific (Lenexa, KS). All other data were generated using CLSI reference BMD or agar dilution (15) or using EUCAST reference methods (16).

### Bacterial isolates

A selection of clinical isolates of *A. urinae* (*n* = 117), *A. viridans* (*n* = 29), and *A. sanguinicola* (*n* = 29) was obtained from three laboratories across the USA: Medical College of Wisconsin, ARUP Laboratories, and Vanderbilt University Medical Center. Isolates were identified by submitting laboratory’s routine standard-of-care methods; identification was confirmed by MALDI-TOF MS (Bruker Daltonics) for any isolates that did not demonstrate colony morphology consistent with *Aerococcus* spp. Isolates were restruck to tryptic soy agar with 5% sheep’s blood (BD, Sparks, MD) upon receipt and stored at −70°C in tryptic soy broth with 20% glycerol (Hardy Diagnostics, Santa Ana, CA). Isolates were subcultured twice from frozen stocks prior to testing.

### Broth microdilution

BMD was performed according to CLSI M07 guidelines (20). Briefly, 96-well panels were prepared in-house with the HTF plate dispenser system (MDZ Automation, Chicago, IL). Each panel included the antimicrobial agents amoxicillin, ampicillin, ceftriaxone, cefotaxime, ciprofloxacin, levofloxacin, meropenem, nitrofurantoin, penicillin, tetracycline, trimethoprim-sulfamethoxazole (SXT), and vancomycin in a twofold dilution series in cation-adjusted Mueller-Hinton broth (CA-MHB) supplemented with 5% lysed horse blood (LHB; Sigma Aldrich, St. Louis, MO). All antimicrobial agents were obtained from US Pharmacopeia (Rockville, MD). Preliminary experiments showed poor growth of isolates in panels made with BBL CA-MHB, so panels were remade with Difco CA-MHB (BD). A BBL CA-MHB growth control well was included in each panel, in addition to the Difco CA-MHB growth control well. *Streptococcus pneumoniae* ATCC 46919 was used for quality control testing of the panels each day of testing. Plates were incubated at 35°C in 5% CO_2_ for 20–24 h and read independently by two readers. If discernable growth was not present in the growth control well(s), panels were re-incubated up to a final 44 h incubation.

### Disk diffusion testing

Disk diffusion was performed according to CLSI M02 (21), using blood Mueller-Hinton agar plates (BMHA) from Remel (Lenexa, KS) or BD. Disks were obtained from BD and included ampicillin (2 µg), ceftriaxone (30 µg), cefotaxime (30 µg), ciprofloxacin (5 µg), levofloxacin (5 µg), meropenem (10 µg), penicillin (10 U), nitrofurantoin (300 µg), SXT (1.25 µg trimethoprim, 23.75 µg sulfamethoxazole), tetracycline (30 µg), and vancomycin (30 µg). Disk diffusion was performed using the same inoculum used for BMD, such that the two tests were performed in parallel. Disk diffusion zones of growth inhibition (ZOI) were evaluated by two independent readers; any fuzzy ZOI or double ZOI were noted in study records. *S. pneumoniae* ATCC 49619 was used for quality control each day of testing*,* which was consistently within the mid-range of CLSI QC standards.

### Data analysis

Disk diffusion results were compared to MICs generated from reference BMD. Categorical agreement, very major errors (VME), major errors (ME), and minor errors (MI) were evaluated using the error rate-bound method (22). MIC breakpoints used for this assessment are listed in Table 2. Best-fit disk diffusion breakpoint estimates were generated from disk-to-MIC correlates using the diffusion Breakpoint Estimation Testing Software (version 1.5; https://dbets.shinyapps.io/dBETS/). Discordant results between disk diffusion and BMD interpretations were categorized as follows: VME, susceptible by disk diffusion and resistant by BMD; ME, resistant by disk diffusion and susceptible by BMD; and MI, a discrepancy between disk diffusion and BMD involving an intermediate result. Acceptable VME, ME, and MI error rates for isolates in the *I ±* 1 category with BMD MIC values within 1 log_2_ dilution of the intermediate breakpoint were <10%, <10%, and <40%, respectively (22). For isolates in the <*I* − 2 or >*I* + 2 categories with BMD MIC values outside one doubling dilution of the intermediate breakpoint, acceptable VME, ME, and MI error rates were <2%, <2%, and <5%, respectively.

**TABLE 2:** Clinical and Laboratory Standards Institute M45 fourth edition tentative clinical breakpoints for *Aerococcus* species

	MIC interpretation (µg/mL)			Disk diffusion interpretation (mm)		
Antimicrobial agent	Susceptible	Intermediate	Resistant	Susceptible	Intermediate	Resistant
Penicillin^*a*^	≤0.12	0.25	≥0.5	≥31	27–30	≤26
Ampicillin^*a*^	≤0.25	0.5	≥1	≥23	18–22	≤17
Cefotaxime	≤1	2	≥4	None established		
Ceftriaxone	≤1	2	≥4	None established		
Meropenem	≤0.5	–^*b*^	–^*b*^	None established		
Vancomycin	≤1	–^*b*^	–^*b*^	≥19	–^*b*^	–^*b*^
Ciprofloxacin	≤1	2	≥4	≥21	18–20	≤17
Levofloxacin	≤2	4	≥8	≥17	14–16	≤13
Nitrofurantoin^*a*^	≤32	–^*b*^	≥64	≥17	–^*b*^	≤16
Tetracycline	≤2	4	≥8	≥23	19–22	≤18

^*a*^ Breakpoints were updated in the M45 fourth edition.

^*b*^ –, indicates no breakpoint.

For MICs, essential agreement was defined as the same MIC, within the accepted variability of BMD, which is ±1 log_2_ dilution. Epidemiological cut-off values (ECVs) were assessed using CLSI methods but did not fully meet data requirements outlined in M23 and were considered estimates only (22). Specifically, the number of testing laboratories (*n* = 3 or more per M23) and availability of endpoint MICs were not met.

## RESULTS

### Bacterial growth in AST media

Ten challenge isolates (five *A*. *urinae*, three *A*. *sanguinicola,* and two *A*. *viridans*) were tested by disk diffusion on Remel and BD BMHA in parallel, using the same inoculum. Growth was visibly heavier on BD plates (Fig. S1), but median ZOI were within 1 mm for each isolate and antimicrobial agent tested between the two brands of BMHA (data not shown). Remel BMHA was used for the remainder of the study as this was the medium most consistently available during the study period due to COVID-19 pandemic shortages. All isolates grew well within 24 h on this medium.

In contrast, growth in BMD panels was dependent on the brand of CA-MHB that was supplemented with 5% lysed horse blood, even when incubation was extended to 44 h. Among *A. urinae*, 100/117 (85.4%) isolates grew in Difco media, 85 at 24 h and 15 at 44 h (Table 3). In contrast, only 58/117 (49.6%) *A*. *urinae* grew in BBL media, 46 at 24 h and 12 at 44 h. All 29 isolates of *A. sanguinicola* grew in Difco media, and 28 of these isolates grew within 24 h. One isolate (3.4%) of *A. sanguinicola* did not grow in BBL media, and one (3.4%) took 44 h. Finally, all 29 *A*. *viridans* grew well in both Difco and BBL media at 24 h (Table 3).

**TABLE 3:** Growth of *Aerococcus* isolates in BD and Difco brand cation-adjusted Mueller-Hinton broth supplemented with 5% lysed horse blood^*a*^

Species	*N*	Difco CA-MHB + 5% LHB		BD CA-MHB + 5% LHB	
		Growth at 24 h	Growth at 44 h	Growth at 24 h	Growth at 44 h
*Aerococcus sanguinicola*	29	96.6%	100.0%	93.1%	96.6%
*Aerococcus urinae*	117	72.6%	85.4%	10.2%	49.6%
*Aerococcus viridans*	29	100.0%	100.0%	100.0%	100.0%

^*a*^ CA-MHB + 5% LHB, CA-MHB supplemented with 5% lysed horse blood.

### Update to penicillin breakpoints

The tentative ECV for penicillin was calculated using data from 3,078 isolates of *A. urinae*, which showed a modal MIC of ≤0.06 µg/mL (87.8% of isolates). Data were only available from three sources for *A. sanguinicola* (*n* = 668 isolates) and *A. viridans* (*n* = 580 isolates), which also showed modal MIC of ≤0.06 µg/mL for these species. MIC distributions evaluated are shown in Fig. S2 and compared to EUCAST distributions published online (www.eucast.org). A definitive ECV could not be established, as the majority of data (>75% for each isolate) came from a single laboratory, which performed testing using lyophilized panels, and endpoint MICs were not available for most isolates.

### Correlation between ampicillin and amoxicillin MICs

Prior editions of the CLSI M45 did not include ampicillin or amoxicillin breakpoints, although the aminopenicillins are used more frequently than penicillin in many countries, particularly amoxicillin for treatment of UTI. Generally, amoxicillin and ampicillin are equivalent agents, with ampicillin serving as a surrogate for amoxicillin in M100 for *Enterococcus* spp., *Haemophilus influenzae,* and *Haemophilus parainfluenzae* (23). We evaluated the MIC agreement between ampicillin and amoxicillin for the isolates in this study. Among the 158 isolates, essential agreement of MICs was achieved for 153/158 isolates (96.8%). No systematic difference between ampicillin and amoxicillin MICs was noted. Three isolates had an ampicillin MIC of ≤0.06 µg/mL (one *A*. *sanguinicola* and two *A*. *urinae*) but an amoxicillin MIC of 0.25 µg/mL, although both results are susceptible. One isolate of *A. urinae* had an ampicillin MIC of 1 µg/mL (resistant) and amoxicillin MIC of ≤0.125 µg/mL (susceptible), and one isolate of *A. urinae* had an ampicillin MIC of 0.5 µg/mL (intermediate) and amoxicillin MIC of ≤0.125 µg/mL (susceptible). Based on these data, the committee felt that ampicillin results could reasonably be used to predict amoxicillin results, with the risk of an occasional over-calling of resistance when using ampicillin.

### Establishment of nitrofurantoin MIC breakpoints

The modal nitrofurantoin MIC for *A. urinae* was ≤1 µg/mL, 8 µg/mL for *A. sanguinicola,* and >32 µg/mL for *A. viridans*, based on data from 2 (*n* = 129 isolates), 1 (*n* = 29 isolates), and 1 (*n* = 29 isolates) laboratories, respectively (Fig. S4). Very little additional data were available to rationalize breakpoints. CLSI Enterobacterales, *Staphylococcus* spp., and *Enterococcus* spp. breakpoints published in M100 are ≤32 µg/mL (susceptible), 64 µg/mL (intermediate), and ≥128 µg/mL (resistant). These breakpoints have not been updated since their first publication in the 1960s and pharmacodynamic parameters for nitrofurantoin have not been determined for any species. One study prospectively evaluated the clinical and microbiological outcomes for patients with UTI caused by *A. urinae* (*n* = 50) and *A. sanguinicola* (*n* = 19). Clinical success for *A. urinae* was 71% when treated with nitrofurantoin vs 100% when treated with ciprofloxacin, cefadroxil, pivmecillinam, or amoxicillin. Similarly, clinical success was 50% for *A. sanguinicola* treated with nitrofurantoin vs 100% for cefadroxil or amoxicillin. In this study, it was unclear if the failures were due to *Aerococcus* spp., as six cases of clinical failure grew a different uropathogen at test of cure, and MICs (measured by gradient diffusion) were not a significant predictor of outcome (4). Susceptible breakpoints were chosen to align with other CLSI criteria (susceptible, ≤32 µg/mL). Due to the absence of data for isolates with MICs >32 µg/mL, the resistant breakpoint was set at ≥64 µ/mL.

MIC breakpoints for all other antimicrobial agents listed in Table 2 were reviewed against contemporary MIC data, and no other changes were made (data not shown).

### Disk diffusion correlates

BMD and disk diffusion were performed on a collection of 158 isolates (*n* = 100 *A*. *urinae*, 29 *A*. *sanguinicola,* and 29 *A*. *viridans*) to establish disk diffusion breakpoints. Disk diffusion correlates to MIC were assessed using the new, fourth edition M45 breakpoints (Table 4), by testing isolates by disk diffusion and BMD in parallel, using the same inoculum. Scatterplots for established disk correlates are in Fig. S4. For penicillin, the median ZOI surrounding a 10 U penicillin disk was 38 mm (range, 6–70 mm). Best-fit disk diffusion correlates to penicillin MIC breakpoints (Table 2) were ≥31 mm (susceptible), 27–30 mm (intermediate), and ≤26 mm (resistant). Two VME were noted, both with MICs of 0.5 µg/mL and ZOI of 35 mm (*A. viridans*) and 33 mm (*A. urinae*). A single ME was observed for an isolate of *A. sanguinicola* with an MIC of 0.06 µg/mL and a ZOI of 26 mm. These errors were within M23 acceptance criteria, using the error rate-bound method. In contrast, MI were slightly above the acceptable 5.0% for ≤*I* + 2, at 5.3% for isolates with MICs ≤0.06 µg/mL and ZOI in the intermediate category. Two isolates had ZOI of 29 mm (one *A*. *urinae* and one *A*. *sanguinicola*) and four of 30 mm (all *A. sanguinicola*). Adjusting the disk breakpoints led to unacceptable rates of VME and ME (data not shown). Of note, among the 29 *A*. *sanguinicola* isolates evaluated, 5 MI (all toward a more resistant interpretation) and 1 ME were observed, suggesting the disk correlates may overcall resistance for this species. However, adjusting these disk correlates did not improve performance for *A. sanguinicola* (data not shown).

**TABLE 4:** Disk diffusion correlate to MIC results, for breakpoints listed in Table 2^*a*^

Antimicrobial agent (S/I/R disk breakpoint in millimeters)		MIC range	Number of isolates	Very major error (%)	Major error (%)	Minor error (%)
Penicillin (≥31/27–30/≤26)	≥*I* + 2	≥1	8	0 (0)	–^*c*^	0 (0)
	*I* + 1 to *I* − 1	0.12–0.5	37	2 (5.4)	0 (0)	10 (27.0)
	≤*I* + 2	≤0.06	113	–^*c*^	1 (0.88)	**6 (5.3)**
		Total	158	2 (10.5)	1 (0.81)	16 (10.1)
Ampicillin (≥23/18–22/≤17)	≥*I* + 2	≥2	2	0	–^*c*^	0
	*I* + 1 to *I* − 1	0.25–1	16	1 (6.3)	1 (6.3)	5 (31.3)
	≤*I* + 2	≤0.125	140	–^*c*^	0	1 (0.7)
		Total	158	1 (16.7)	1 (0.68)	6 (3.8)
Ceftriaxone (≥26/18–25/≤17)	≥*I* + 2	≥8	9	**3 (33.3)**	–^*c*^	**3 (33.3)**
	*I* + 1 to *I* − 1	1–4	47	3 (6.4)	0	**23 (48.9)**
	≤*I* + 2	≤0.5	102	–^*c*^	0	**7 (6.9)**
		Total	158	6 (30.0)	0	33 (20.9)
Cefotaxime (≥27/18–26/≤17)	≥*I* + 2	≥8	6	**1 (16.7)**	–^*c*^	**1 (16.7)**
	*I* + 1 to *I* − 1	1–4	36	2 (5.6)	0	**16 (44.4)**
	≤*I* + 2	≤0.5	116	–^*c*^	0	**10 (8.6)**
		Total	158	3 (23.1)	0	27 (17.1)
Nitrofurantoin (≥17/-/≤16)		≥64	32	**2 (6.3)**	–^*c*^	–^*c*^
		≤32	126	–^*c*^	1 (0.70)	–^*c*^
		Total	158	2 (5.4)	1 (0.83)	–^*c*^
Ciprofloxacin (≥21/18–20/≤17)	≥*I* + 2	≥8	25	**2 (8.0)**	–^*c*^	**5 (20.0)**
	*I* + 1 to *I* − 1	1–4	80	2 (2.5)	0	**33 (41.3)**
	≤*I* + 2	≤0.5	53	–^*c*^	0	1 (1.9)
		Total	158	4 (10.0)	0	43 (27.2)
Levofloxacin (≥17/14–16/≤13)	≥*I* + 2	≥16	20	**1 (5.0)**	–^*c*^	0
	*I* + 1 to *I* − 1	2–8	53	1 (1.9)	0	12 (22.6)
	≤*I* + 2	≤1	84	–^*c*^	1 (1.2)	**5 (5.9)**
		Total	157^*b*^	1 (2.5)	1 (0.92)	17 (10.8)
Tetracycline (≥23/19–22/≤18)	≥*I* + 2	≥16	11	0	–^*c*^	0
	*I* + 1 to *I* − 1	2–8	1	0	0	0
	≤*I* + 2	≤1	145	–^*c*^	1 (0.7)	1 (0.7)
		Total	157^*b*^	0	1 (0.68)	1 (0.63)
Vancomycin (≥19/-/-)		≥2	1	0	–^*c*^	–^*c*^
		≤1	157	–^*c*^	1 (0.6)	–^*c*^
		Total	158	0	1 (0.6)	–^*c*^

^*a*^ Bolded values indicate error rates above what is generally acceptable by CLSI.

^*b*^ One isolate had no disk diffusion result due to dropped disk.

^*c*^ –, indicates no breakpoint.

Ampicillin disk diffusion was assessed using a non-CLSI but commercially available 2 µg drug mass, selected for its lower concentration compared to the 10 µg standard CLSI disk. Median ampicillin disk ZOI was 30 mm (range, 6–50 mm, Table 5). Best-fit disk diffusion correlates to the ampicillin MICs were ≥23 mm for susceptible and ≤17 mm for resistant. One VME and one ME for isolates in the 0.25–1 µg/mL range were observed. The VME was for an isolate of *A. urinae* with an ampicillin MIC of 1 µg/mL and a ZOI of 30 mm. The ME was an isolate of *A. viridans* with an MIC of 0.25 µg/mL and ZOI of 11 mm. Five MI were observed for isolates of *A. urinae*, with no trend in the directionality to the errors. In addition, an MI for an isolate of *A. urinae* with an MIC of 0.12 µg/mL and a ZOI of 20 mm was observed.

**TABLE 5:** ZOI ranges for *Aerococcus* spp.

Antimicrobial agent	Disk content	Median ZOI (mm)	Average ZOI (mm)	ZOI range (mm)
Penicillin	10 U	38	37.4	6–70
Ampicillin	2 µg^*a*^	30	30.0	6–50
Cefotaxime	30 µg	33	33.0	6–56
Ceftriaxone	30 µg	30	30.4	6–50
Meropenem	10 µg	37	36.5	20–60
Nitrofurantoin	300 µg	29	27.8	6–47
Ciprofloxacin	5 µg	21	21.5	6–50
Levofloxacin	5 µg	20	19.9	6–46
Tetracycline	30 µg	33	31.8	6–52
Vancomycin	30 µg	26	26.7	18–44
Trimethoprim-sulfamethoxazole	1.25/23.75 µg	6	10.6	6–40

^*a*^ Non-standard CLSI disk.

No acceptable disk diffusion correlates could be determined for ceftriaxone, cefotaxime, and meropenem using standard CLSI disks. Median disk diffusion ZOI were 30 mm (range, 6–50 mm), 33 mm (range, 6–56 mm), and 37 mm (range, 20–60 mm), respectively. Cefotaxime ZOI for resistant isolates (*n* = 13) ranged from 6 to 26 mm, which overlapped with the ZOI measured for isolates with MICs of ≤0.25 µg/mL, which ranged from 20 to 56 mm. For resistant isolates (*n* = 13), 46.2% had ZOI of 22–34 mm, which overlapped with 54.1% of the 135 susceptible isolates. Best-fit disk diffusion correlates led to unacceptable VME rates (Table 4). Similarly, ceftriaxone ZOI for resistant isolates (*n* = 20) ranged from 6 to 36 mm, overlapping with isolates at the lowest MIC of ≤0.12 µg/mL (range of ZOI, 25–50 mm). Over half of the susceptible isolates (*n* = 123, 61.7%) had disk diffusion ZOI of 19–32 mm; 70% of the 20 resistant isolates also had ZOI in the 19–32 mm range. Finally, for meropenem, non-susceptible isolates with MIC of 1 µg/mL had ZOI of 25–42 mm. Three of the 11 non-susceptible isolates (27.3%) had disk zones ≥40 mm. Because of the near-complete overlap of meropenem ZOI for susceptible and resistant isolates, no correlates were attempted.

Median nitrofurantoin ZOI was 29 mm (range, 6–47 mm). Best-fit correlates were ≥17 mm for susceptible and ≤16 mm for resistant. Two VME and one ME result was noted with these correlates, all for isolates of *A. urinae*. The VME were for two isolates with MIC >32 µg/mL and ZOI of 22 and 31 mm, whereas the ME was for an isolate with a ZOI of 16 mm and an MIC of 32 µg/mL. The two VME led to a VME rate of 6.3%, which is above the desired 5% (Table 4).

Median ZOI for ciprofloxacin and levofloxacin were 21 and 20 mm, respectively. Best-fit correlates for ciprofloxacin were ≥21 mm susceptible and ≤17 mm resistant. VME were noted for *A. urinae* and *A. sanguinicola* with MIC >4 µg/mL and ZOI of 35 and 38 mm, respectively, leading to a VME rate of 8.0% for isolates with MIC >4 µg/mL, above M23 guidelines. ME were noted for isolates with MICs of 0.5 µg/mL (an isolate of *A. viridans* with a ZOI of 17 mm) and 1 µg/mL (one isolate of *A. urinae* and one *A*. *viridans* with a ZOI of 14 mm and an isolate of *A. viridans* with a ZOI of 16 mm). In addition, five MI for isolates with MIC >4 µg/mL (20%) and 33 MI for isolates with MIC 1–4 µg/mL (41.3%) were noted. For the latter group of MI, 9 were undercalled (i.e., 4 with a resistant MIC and intermediate ZOI, and 5 with an intermediate MIC and susceptible ZOI), and 24 were overcalled (i.e., 5 with intermediate MIC and resistant ZOI and 19 with a susceptible MIC and intermediate ZOI). Both these MI rates were above the acceptable limits per M23. Best-fit correlates for levofloxacin were ≥17 mm susceptible and ≤13 mm resistant. One ME (*A. sanguinicola* with an MIC 1 µg/mL and ZOI 6 mm) and two VME (both *A. urinae*, one with MIC 8 µg/mL and ZOI 30 mm and one with MIC of >16 µg/mL and ZOI 23 mm) were noted. The one VME for the isolate with MIC of >16 µg/mL resulted in an error rate above M23 guidelines, resulting in a 5.0% VME rate. We attempted to use the levofloxacin disk to predict ciprofloxacin MIC, but this did not yield improved results.

Median tetracycline disk ZOI was 33 mm (range, 6–52 mm, Table 5). Good correlation was achieved for disk diffusion, with a breakpoint of ≥23 mm, susceptible and ≤18 mm, resistant. All errors were within M23 criteria (Table 4). Vancomycin median ZOI was 26 mm (range, 18–44 mm), and best-fit correlates were ≥19 mm, susceptible. Only one isolate was non-susceptible to vancomycin, an isolate of *A. sanguinicola* with a vancomycin MIC of 2 µg/mL and a ZOI of 18 mm. An additional 10 isolates had MICs of 1 µg/mL, with ZOI ranging from 18 mm (a single ME for an isolate of *A. urinae*) to 32 mm.

As expected, the median SXT ZOI was 6 mm (i.e., no ZOI) for *A. urinae* but was also 6 mm for *A. sanguinicola* and *A. viridans*. Modal MICs for these isolates were ≤0.25/4.75, 1/19, and ≤0.25/4.75 µg/mL, respectively. MICs ranged from ≤0.25/4.75 to >8/152 µg/mL across all species, for those that displayed no ZOI surrounding the SXT disk. Furthermore, isolates with MIC ≤0.25/4.75 µg/mL displayed ZOI that ranged from 6 to 40 mm. No disk correlation was possible with these data.

## DISCUSSION

Over the past 2 years, volunteers for CLSI have embarked on a revision of the M45 guideline, including re-assessment of the breakpoints published for *Aerococcus* spp. Two primary changes were requested by users of the document for *Aerococcus* spp.: (i) increased oral options for the treatment of aerococcal UTI and (ii) addition of disk diffusion criteria, which would enable testing in more clinical laboratories that do not have access to reference BMD methods.

We encountered significant growth issues for *A. urinae* by BMD, which we found to be related to the brand of CA-MHB base used. EUCAST identified similar growth challenges for *A. urinae* using BMD panels prepared using EUCAST Mueller-Hinton fastidious medium. Difco base CA-MHB was used for media preparation in that study (Erika Matuschek, personal communication to R.H.), and 18% of *A. urinae* required 44 h of incubation to demonstrate sufficient growth to read MIC values (24). In contrast, we found 14.6% of *A. urinae* did not grow in Difco CA-MHB + LHB, even at 44 h growth, and over half did not grow in BBL CA-MHB base (Table 3). We have noted issues in the ability of MHA from different manufacturers to support the growth of fastidious micro-organisms in other studies (25). The reasons for these discrepancies are unclear, as all brands of CA-MHB and MHA manufactured for use in the USA meet technical standards put forth by the International Standard Organization for these media (26). When developing AST standards, CLSI now requires evaluation across brands and lots of media to help recognize these differences earlier in the development process (22). Nonetheless, clinical laboratories should be cognizant of the media- and brand-based differences in growth and always ensure growth is sufficient to evaluate before reading AST results by both disk and MIC methods. The CLSI revision to M45 will include guidance for incubation up to 44 h. Interestingly, in our study, all species grew very well on BMHA, including those that did not grow by BMD (Table 3). Kling and colleagues also found agar dilution and gradient diffusion tests on BMHA were easier to read for *A. urinae* than BMD methods (19). We did see differences in growth on BMHA across manufacturers of BMHA, but both brands evaluated supported growth sufficiently to read ZOIs. It is possible that agar-based methods, such as disk diffusion, agar dilution, and perhaps gradient diffusion, are better methods for evaluating the antimicrobial agent susceptibility of aerococci and, *A. urinae,* in particular. This should be further evaluated in future studies.

CLSI and EUCAST have harmonized optimal criteria for the evaluation of drug masses for disk diffusion tests (27). An optimal drug mass should yield ZOI for wild-type bacteria in the range of 15–35 mm, and ideally none above 30 mm. In addition, a 2–3 mm increase in zone diameters for each log_2_ decrease in MIC should be demonstrated for non-wild-type isolates (27). These parameters help ensure that there is reproducible and reliable separation between isolates belonging to different interpretive categories determined by MIC. Drug masses that are overly high lead to very large ZOI, and those that are too low lead to very small ZOI, both of which can make differentiation of susceptible and resistant isolates challenging. The median ZOI for penicillin, cefotaxime, meropenem, and tetracycline were >30 mm. Despite this, reasonable disk diffusion correlates to the MIC interpretations were possible for penicillin, ampicillin, nitrofurantoin, ciprofloxacin, levofloxacin, tetracycline, and vancomycin (Table 4). No correlates could be determined for cefotaxime, ceftriaxone, or meropenem. No improvement in performance was possible when the data were evaluated by species (not shown), and the revision in breakpoints ensured that MIC breakpoints did not split MIC wild types, which would be expected to result in higher errors. In addition, disk correlates could not be established for SXT, which is likely related to the sheep blood vs horse blood used in disk diffusion vs BMD tests, as discussed (28). Challenges with disk diffusion testing of beta-lactams for fastidious bacteria have been encountered previously. When penicillin resistance was first encountered among the pneumococci, it was shown that resistant isolates demonstrated large ZOI, and a breakpoint of ≤35 mm was required to reliably detect resistance using a 10 U penicillin disk (6 µg equivalent). However, 22% of isolates susceptible to penicillin by MIC also yielded ZOI ≤35 mm (29). A follow-up study in South Africa found that titration of penicillin disk potency from 6 µg (10 U) to 0.15 µg led to stepwise improvements in separation of penicillin-resistant and -susceptible isolates. Reduction of the potency below 0.15 µg led to poor resolution with small ZOI sizes for both resistant and susceptible isolates that could not be distinguished (30). For this reason, the 1 µg surrogate oxacillin disk was introduced for testing pneumococci (29). Similarly, it was quickly recognized that disk diffusion using standard 30 µg potency could not be used to separate cefotaxime-susceptible and -resistant isolates of pneumococci (31). Reduction of the drug mass to 1 µg led to improved resolution of resistant from susceptible isolates (32), but this disk has never been commercially produced. Rather, laboratories are instructed to perform MIC methods when testing pneumococci against ceftriaxone and cefotaxime (23). Generally, both CLSI and EUCAST prefer the use of a single drug mass to be used with all relevant species to minimize the complexity of maintaining multiple disks in the laboratory and the risk of testing errors (27). Alternative disk potencies may be considered when absolutely necessary, but even if such disks were developed for the aerococci, it is unlikely these would be manufactured commercially for relatively rare micro-organisms as such development is costly. Furthermore, the US Food and Drug Administration (FDA) does not currently recognize any breakpoints for *Aerococcus* spp., meaning there would be no regulatory pathway to market disks for aerococci in the USA (33). As such, if a serious infection caused by *Aerococcus* spp. is encountered, testing ceftriaxone, cefotaxime, or meropenem should be done using an MIC method, ideally by reference BMD or agar dilution protocols.

While the disk diffusion correlates for penicillin, ampicillin, nitrofurantoin, ciprofloxacin, and levofloxacin did not quite meet M23 standards, the committee felt the directionality of errors (generally minor errors overcalled as resistant by disk diffusion) was reassuring, particularly as disk diffusion testing may be more robust for *A. urinae*. VME identified were only for one to four isolates, and high rates of susceptibility reflected the low number of isolates with elevated MICs evaluated in this study.

We have previously investigated the impact of test medium on *A. urinae* susceptibility to SXT (28). Thymidine concentrations above 0.03 µg/mL inhibit the activity of sulfonamides and trimethoprim. Endogenous thymidine phosphorylase in horse erythrocytes leads to exceedingly low concentrations of thymidine in CA-MHB + 5% lysed horse blood. In contrast, sheep’s blood is thymidine rich, leading to SXT resistance when *A. urinae* is tested on this medium, and poor correlations between MICs determined in lysed horse blood with ZOI determined on BMHA. *A. urinae* harbor an intrinsic *folT* gene, which encodes a folate transport-binding protein (34). As such, it is not surprising to see poor correlation of SXT disk diffusion and BMD results. However, we were surprised to find similar trends toward no ZOI by disk diffusion but low MICs for isolates of *A. viridans* and *A. sanguinicola*. Because of ongoing uncertainty regarding the clinical relevance of this phenotype for aerococci, the CLSI M45 committee chose to eliminate SXT breakpoints for *Aerococcus* spp. Similarly, EUCAST does not publish SXT breakpoints for these micro-organisms (16).

In summary, we present the data used by the volunteers tasked by CLSI to revise *Aerococcus* breakpoints in the M45 guideline. Significant additions include new disk diffusion correlates, addition of new breakpoints for nitrofurantoin and ampicillin, elimination of breakpoints for SXT, and re-assessment of breakpoints for penicillin.

## SUPPLEMENTAL MATERIAL

Figure S1 jcm.00115-25-s0001.pdf Growth of *Aerococcus urinae* on BD (Sparks, MD).

Figure S2 and S3 jcm.00115-25-s0002.pdf Fig. S2: Penicillin MIC results for Aerococcus urinae (n=3078), A. sanguinocola (n=668) and A. viridans (n=580). Fig. S3: Nitrofurantoin MIC results for Aerococcus urinae (n=129), A. sanguinocola (n=29) and A. viridans (n=29)

Fig S3 jcm.00115-25-s0003.pdf Disk diffusion zones of growth inhibition (ZOI) versus MIC for *Aerococcus* tested in this study.

ASM does not own the copyrights to Supplemental Material that may be linked to, or accessed through, an article. The authors have granted ASM a non-exclusive, world-wide license to publish the Supplemental Material files. Please contact the corresponding author directly for reuse.
