# Supplementary material for: Development of disk diffusion susceptibility test methods for Aerococcus spp. and updates to Clinical and Laboratory Standards Institute MIC breakpoints
Source: J Clin Microbiol. 2025 May 14;63(6):e00115-25. doi: 10.1128/jcm.00115-25 (PMC12153260; doi:10.1128/jcm.00115-25)
Supplement: Figure S1 — Growth of Aerococcus urinae on BD (Sparks, MD). [file jcm.00115-25-s0001.pdf]

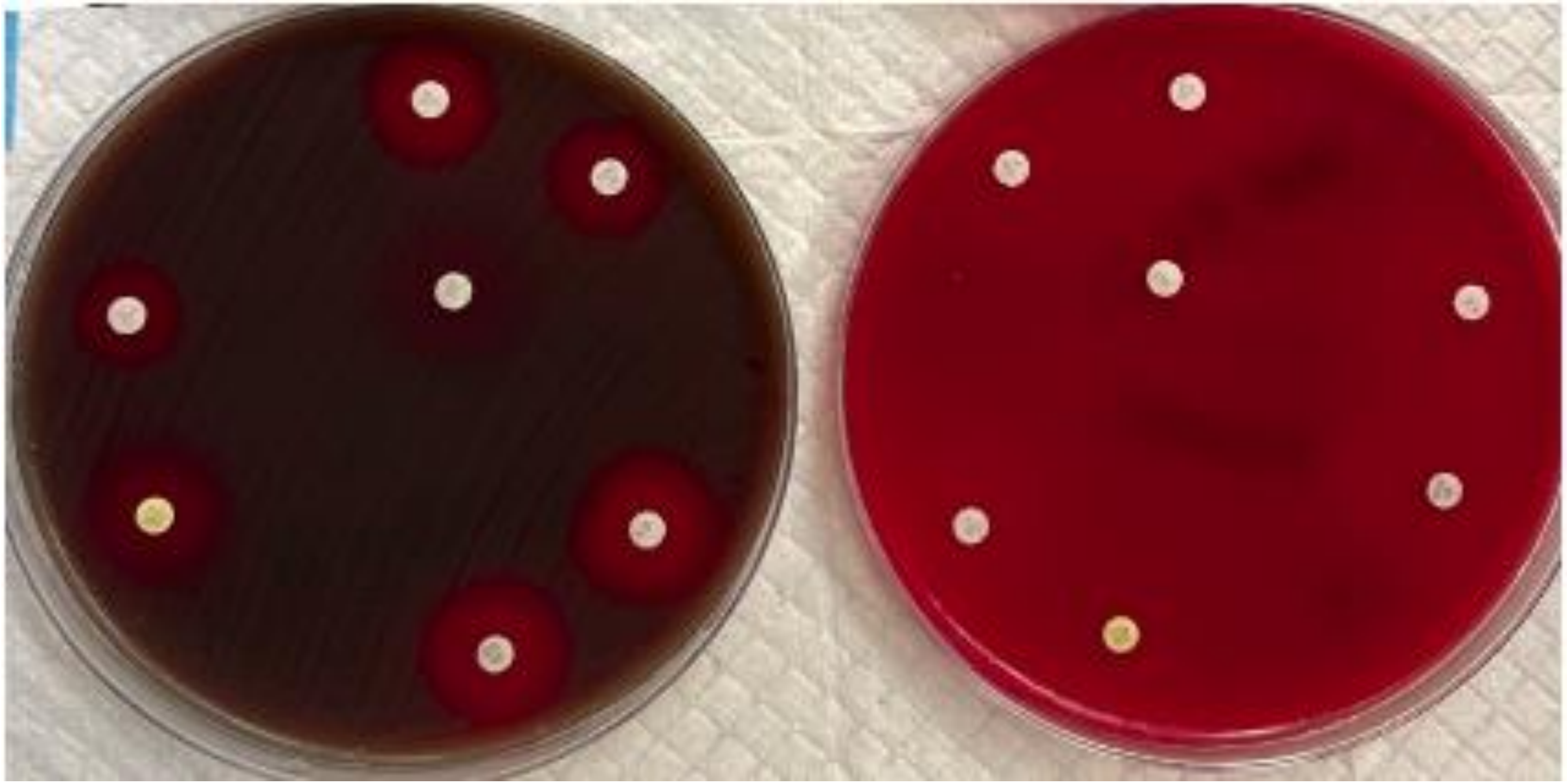

Supplemental Figure 1. Growth of *Aerococcus urinae* on BD (Sparks, MD) Blood Mueller Hinton Agar (left) and Remel (Lenexa, KS) BMHA (right)
