# Supplementary material for: Development of disk diffusion susceptibility test methods for Aerococcus spp. and updates to Clinical and Laboratory Standards Institute MIC breakpoints
Source: J Clin Microbiol. 2025 May 14;63(6):e00115-25. doi: 10.1128/jcm.00115-25 (PMC12153260; doi:10.1128/jcm.00115-25)
Supplement: Figure S2 and S3 — Fig. S2: Penicillin MIC results for Aerococcus urinae (n=3078), A. sanguinocola (n=668) and A. viridans (n=580). Fig. S3: Nitrofurantoin MIC results for Aerococcus urinae (n=129), A. sanguinocola (n=29) and A. viridans (n=29) [file jcm.00115-25-s0002.pdf]

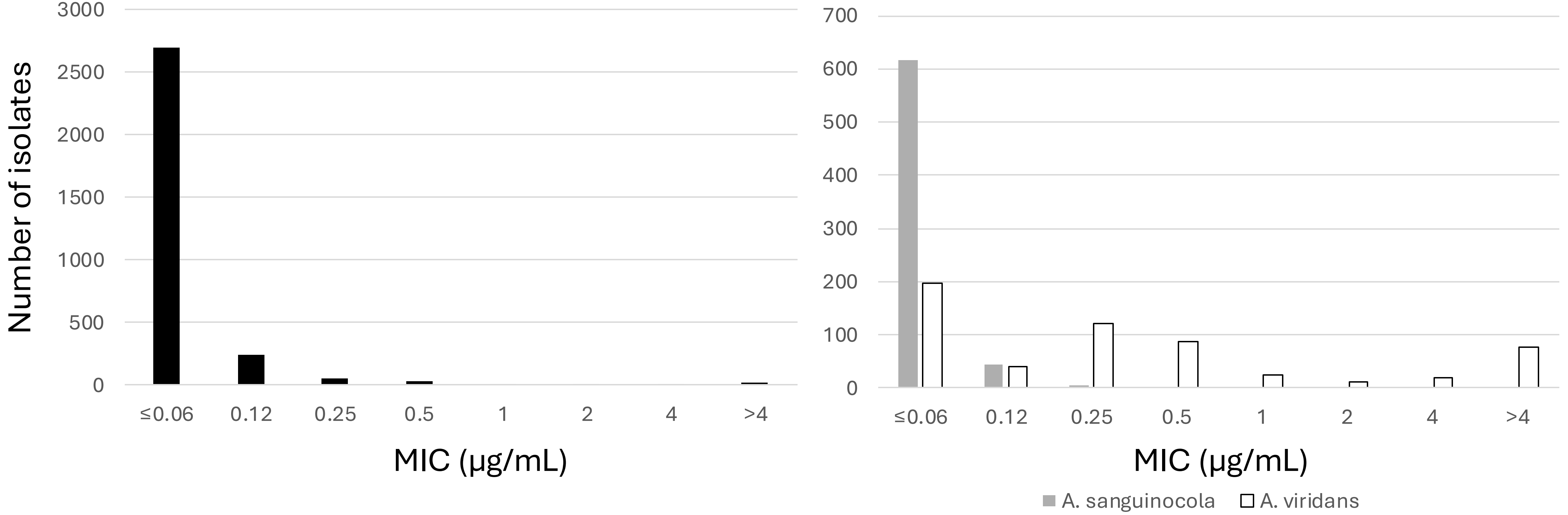

Supplemental Figure 2. Penicillin MIC results for *Aerococcus urinae* (n=3078), *A. sanguinocola* (n=668) and *A. viridans* (n=580)

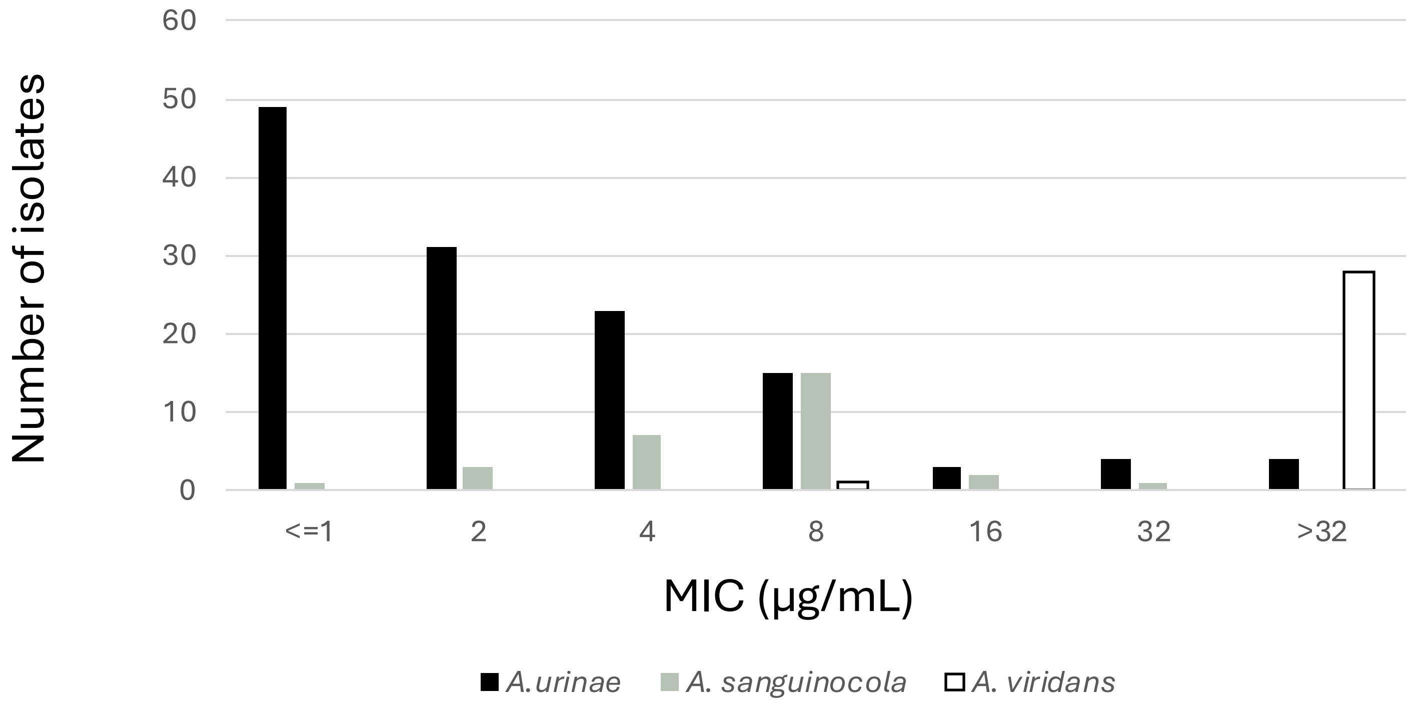

Supplemental Figure 3. Nitrofurantoin MIC results for *Aerococcus urinae* (n=129), *A. sanguinocola* (n=29) and *A. viridans* (n=29)
