# Supplementary material for: Development of disk diffusion susceptibility test methods for Aerococcus spp. and updates to Clinical and Laboratory Standards Institute MIC breakpoints
Source: J Clin Microbiol. 2025 May 14;63(6):e00115-25. doi: 10.1128/jcm.00115-25 (PMC12153260; doi:10.1128/jcm.00115-25)
Supplement: Fig S3 — Disk diffusion zones of growth inhibition (ZOI) versus MIC for Aerococcus tested in this study. [file jcm.00115-25-s0003.pdf]

Supplemental Figure 4. Disk diffusion zones of growth inhibition (ZOI) versus MIC for *Aerococcus* tested in this study

Disk ZOI (mm)

MIC (µg/mL)	6	7	8	9	10	11	12	13	14	15	16	17	18	19	20	21	22	23	24	25	26	27	28	29	30	31	32	33	34	35	36	37	38	39	40	41	42	43	44	≥45
≤0.06																					1			2	4	3	3	3	2	2	13	4	5	2	15	4	11	3	7	29
0.12																											1	1		1			3		1		1		1	2
0.25																	1		1	1	1	1	1	2	5					1			1							
0.5																1	1		1	1	1	1	1	1	1			**1**		**1**										
1	1																																							
2							1					1					1																							
4	1													1	1	1																								
≥8																																								

a. Penicillin (10U disk)

Disk ZOI (mm)

MIC (µg/mL)	6	7	8	9	10	11	12	13	14	15	16	17	18	19	20	21	22	23	24	25	26	27	28	29	30	31	32	33	34	35	36	37	38	39	≥40
≤0.06																		4	6	5	10	4	10	4	16	6	12	4	6	4	6	3	2	1	12
0.12															1					2	4	2	1	2	4	1	3	1		2	1	1			
0.25						1												1	1				1		1							1			1
0.5								1							2						1				1										
1					1										2										1										
2	1								1																										
4																																			
≥8																																			

b. Ampicillin (2 µg disk)

Disk ZOI (mm)

MIC (µg/mL)	6	7	8	9	10	11	12	13	14	15	16	17	18	19	20	21	22	23	24	25	26	27	28	29	30	31	32	33	34	35	36	>36
≤0.12															1		2							1							1	
0.25														4	8	5	6	6	3	9	8	3	5	1		1	2	1		1		
0.5																	4		1	5	13	6	6	8	21	3	5	1	1	3	1	1
1													1		1		1				2			1	2	1	1					
≥2													1																			

c. Vancomycin (30 µg disk)

Disk ZOI (mm)

MIC (µg/mL)	6	7	8	9	10	11	12	13	14	15	16	17	18	19	20	21	22	23	24	25	26	27	28	29	30	31	32	33	34	35	>35
≤0.12																1					1		1	2		1		1			2
0.25																2				1	4		2	2	2	3	1	2		2	3
0.5												1				2	1		3	1	2	1	6		2		1				
1									2		1		4	4	11	8	6	2	1	3			2			1		1			1
2				1					1	2	1		4	2	2	1	1	1					2								
4			3			1			2		3		1	2	1			1					1								
>4	16		2						1		1	1	1		1															1	1

d. Ciprofloxacin (5 µg disk)

Disk ZOI (mm)

MIC (µg/mL)	6	7	8	9	10	11	12	13	14	15	16	17	18	19	20	21	22	23	24	25	26	27	28	29	30	31	32	33	34	35	>35
≤0.03																												1			
0.06																				1											2
0.12																									1						
0.2																						1									
0.25														1				2		1	1	1	3	1	1		1		1		1
0.5									1							1		3	2	1	2		3		4	1					3
1	1								1	1	2		4	6	3	3	3	1	2	1	5	1	4	1	1	1	2				
2										1	3	2	5	6	4			1	1		1								1		
4					3		1		1	1															1				1		
8	3		1	1	2	3	5	2	1	1															1						
16	6							1																							
≥32	12																	1													

e. Levofloxacin(5 µg disk)

Disk ZOI (mm)

MIC (µg/mL)	6	7	8	9	10	11	12	13	14	15	16	17	18	19	20	21	22	23	24	25	26	27	28	29	30	31	32	33	34	35	>35
≤1																			1		1		1	1	1	4	2	1	1		24
2																	1			3	3		3	1			4			1	9
4																	2			2	2	1	1	1	4	1	2	2	1	1	3
8													1		3		3	1	1	1	8		2	1	2	1	1	2		2	2
16																1			1				1					1		1	
32											1					1								1	1					1	
≥64	11		1	3	2	6	3		3	1							1									1					

f. Nitrofurantoin (300 µg disk)

Disk ZOI (mm)

MIC (µg/mL)	6	7	8	9	10	11	12	13	14	15	16	17	18	19	20	21	22	23	24	25	26	27	28	29	30	31	32	33	34	35	>35
≤0.5							1									1			4	7	5	5	7	10	8	8	12	5	9	13	47
1																														1	2
2																															1
4																															
8																															
≥16	1				1	2	3	1					1															1		1	

g. Tetracycline (30 µg disk)

Disk ZOI (mm)

MIC (µg/mL)	6	7	8	9	10	11	12	13	14	15	16	17	18	19	20	21	22	23	24	25	26	27	28	29	30	31	32	33	34	35	>35
≤0.125																				1	1		2	2	5		1	1	1		14
0.25															1			1		2	4	1	3	4	6	3	4	6	2	2	7
0.5														1				1				1	3	5	4	2	1	1	1	2	6
1															2					3		1		2	5	2	2	3			1
2																		1		2			3		3		1	1		2	2
4	1				1										1	1	1		2	1				1	1		1				
>4	2						1							2						1					1	1					1

h. Ceftriaxone (30 µg disk)

Disk ZOI (mm)

MIC (µg/mL)	6	7	8	9	10	11	12	13	14	15	16	17	18	19	20	21	22	23	24	25	26	27	28	29	30	31	32	33	34	35	>35
≤0.25															1				1	1	3	2	7	3	4	7	5	3	5	7	37
0.5																	1	1	1	1		3		1	2	2	6	2		3	7
1																1		1					2	2		4	1		2	1	5
2																							4				1				5
4	1																1	2			1										2
8	1																			1									1		
>8	3																														

i. Cefotaxime (30 µg disk)

Disk ZOI (mm)

MIC (µg/mL)	6	7	8	9	10	11	12	13	14	15	16	17	18	19	20	21	22	23	24	25	26	27	28	29	30	31	32	33	34	35	>35
<=0.06																								2	1	2		2	4	4	15
0.125																						1			2	1	2	2	2	4	11
0.25																			1	1	2		1	1	2	1	3	2	1	2	13
0.5															1				1		1	2		2	2	1					2
1																				1	1	1	1	1	1						
2																							1								1

j. Meropenem (10 µg disk)

Disk ZOI (mm)

MIC (µg/ml)	6	7	8	9	10	11	12	14	15	16	17	18	19	20	21	22	23	24	25	26	27	28	29	30	31	32	33	34	35	>35
≤0.25						1	2	1		1			2			1	1			2				1		1				2
0.5					1	2	1		1	2	2			2	1				1			1	1							1
1						1	2									1														
2							1							1																
4								1	2																1					
8					1			1			1								1					1						
>8																				1									1	1

k. Trimethoprim-sulfamethoxazole (1.25/23.75 µg disk)
